# Supplementary material for: Application of a Micro Free-Flow Electrophoresis 3D Printed Lab-on-a-Chip for Micro-Nanoparticles Analysis
Source: Nanomaterials (Basel). 2020 Jun 30;10(7):1277. doi: 10.3390/nano10071277 (PMC7408601; doi:10.3390/nano10071277)
Supplement: Supplementary file 1 [file nanomaterials-10-01277-s001.pdf]

nanomaterials

Application of a Micro Free-Flow Electrophoresis 3D printed Lab-on-a-Chip for micro-nanoparticles analysis

Federica Barbaresco [a], Matteo Cocuzza [a,b], Candido Fabrizio Pirri [a] and Simone Luigi Marasso [a,b]

[a] Chilab - Materials and Microsystems Laboratory, DISAT, Politecnico di Torino - Via Lungo Piazza d'Armi 6, IT 10034, Chivasso (Turin), Italy
[b] CNR-IMEM, Parco Area delle Scienze, 37a, IT 43124, Parma, Italy

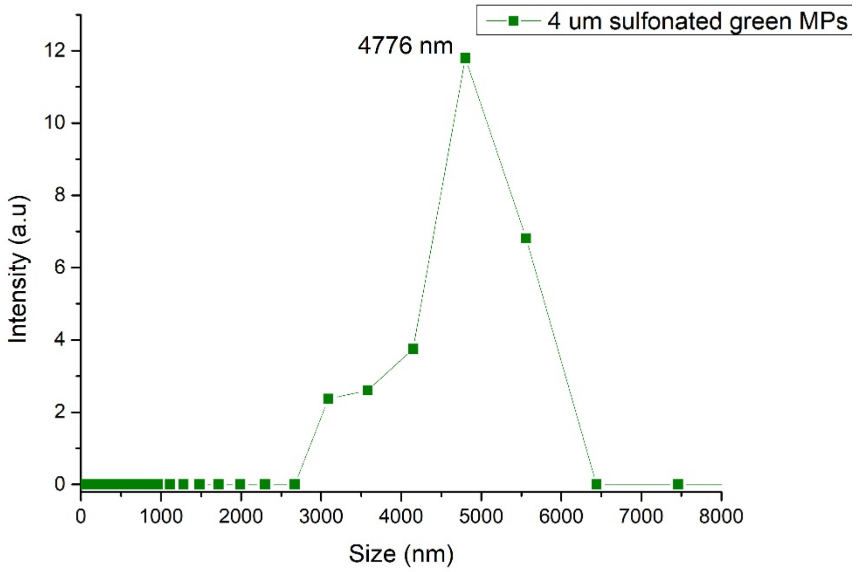

Figure S1. Size measurement of 4 μm MPs by Dynamic Light Scattering.

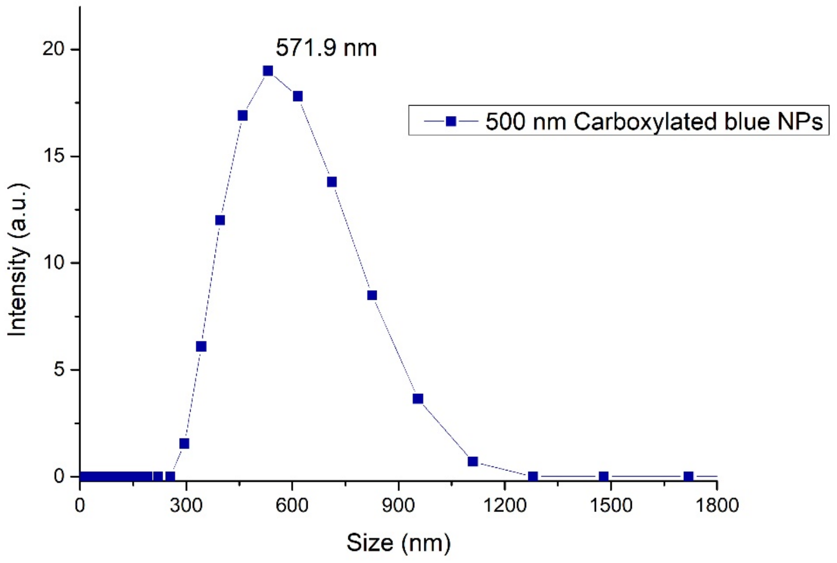

Figure S2. Size measurement of 500 nm NPs by Dynamic Light Scattering.

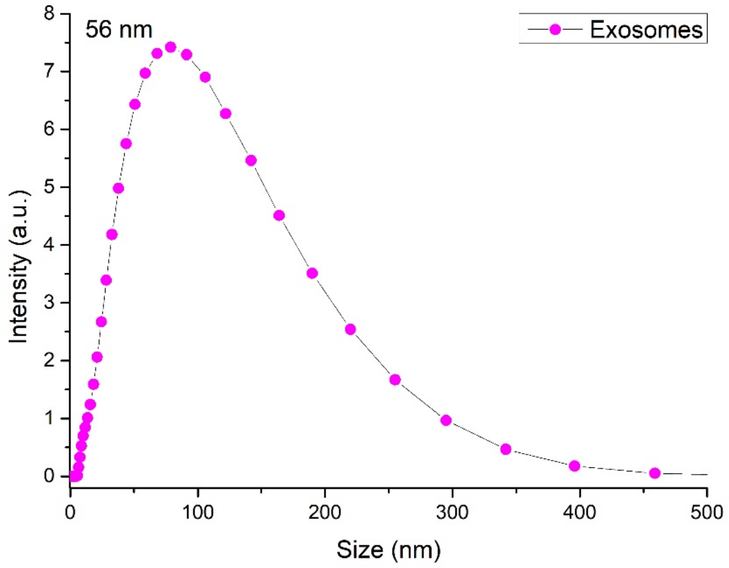

Figure S3. Size measurement of exosomes by Dynamic Light Scattering.

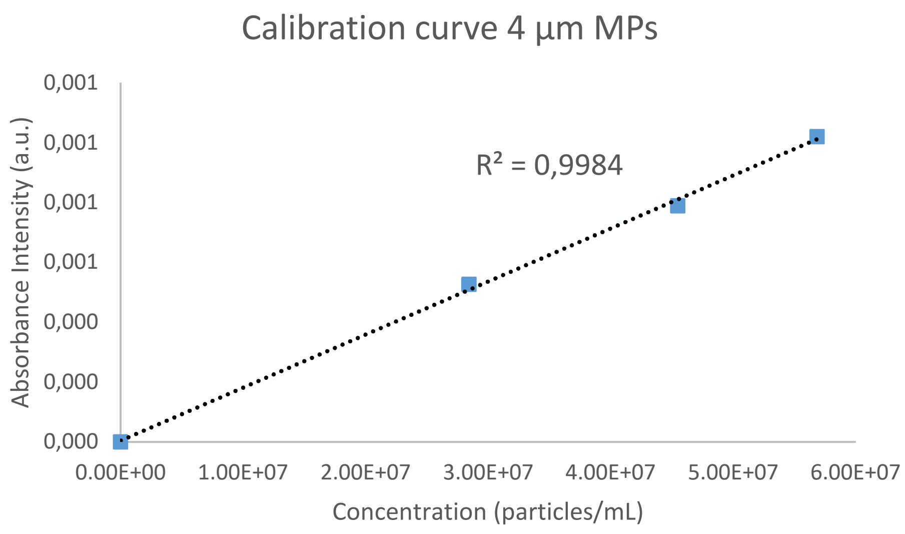

Figure S4. 4 µm MPs calibration curve: trendline equation $y = 2 \times 10^{-8}x + 0.005$.

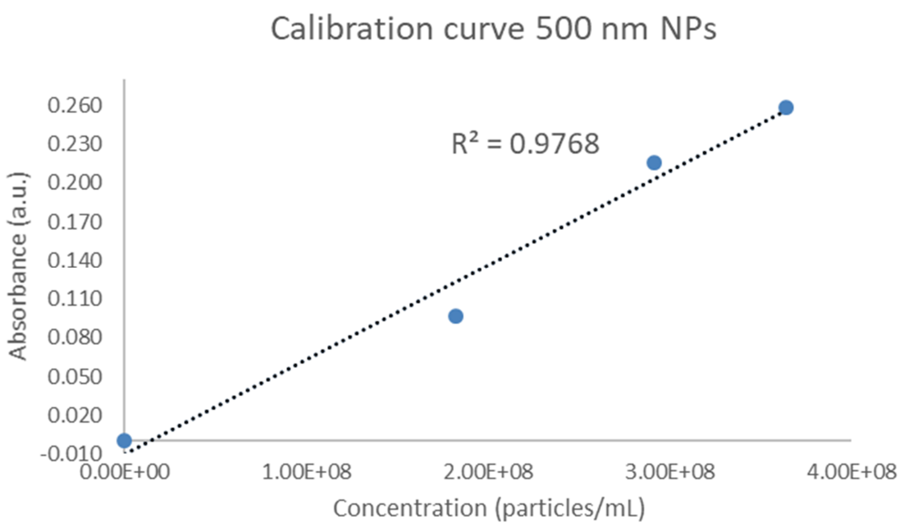

Figure S5. 500 nm NPs calibration curve: trendline equation $y = 7 \times 10^{-10}x - 0.001$

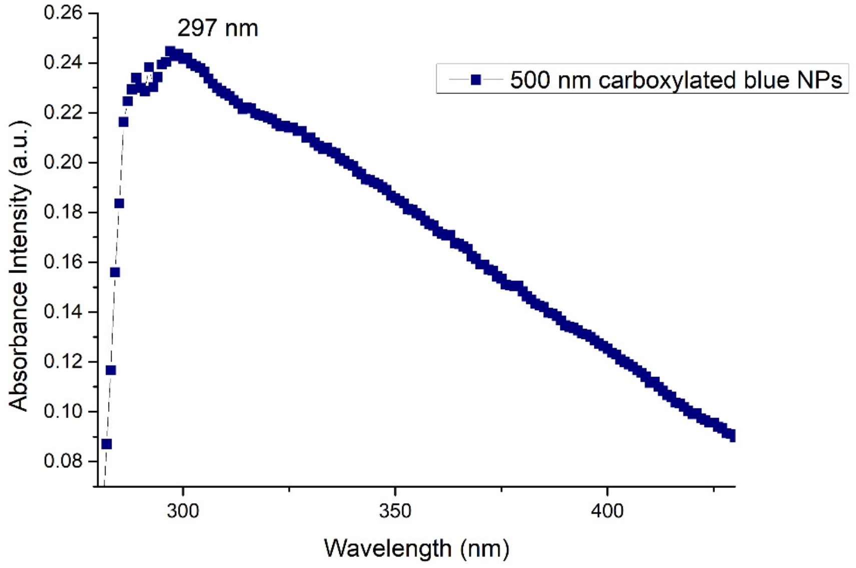

Figure S6. Absorbance spectrum of 500 nm carboxylated NPs: excitation wavelength = 297 nm.

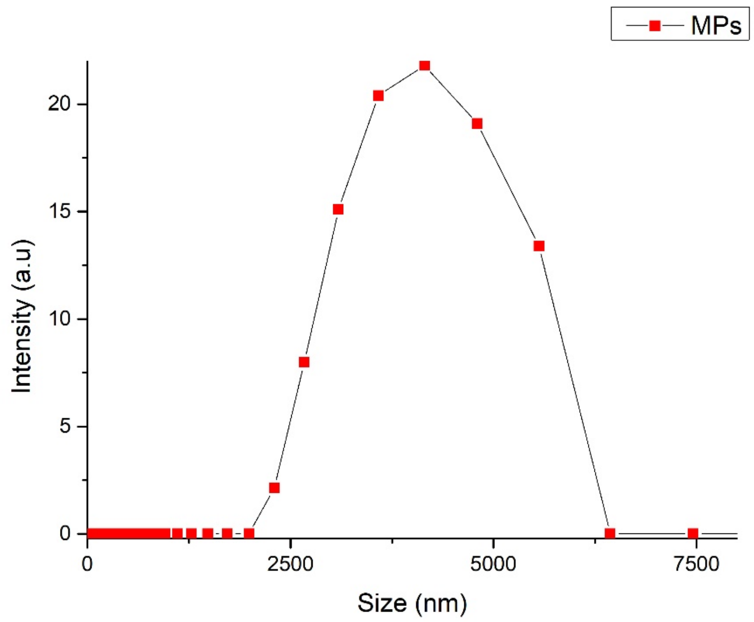

Figure S7. Size measurement of 4 μm MPs by Dynamic Light Scattering at outlet#3 when 0 V were applied at the electrodes.

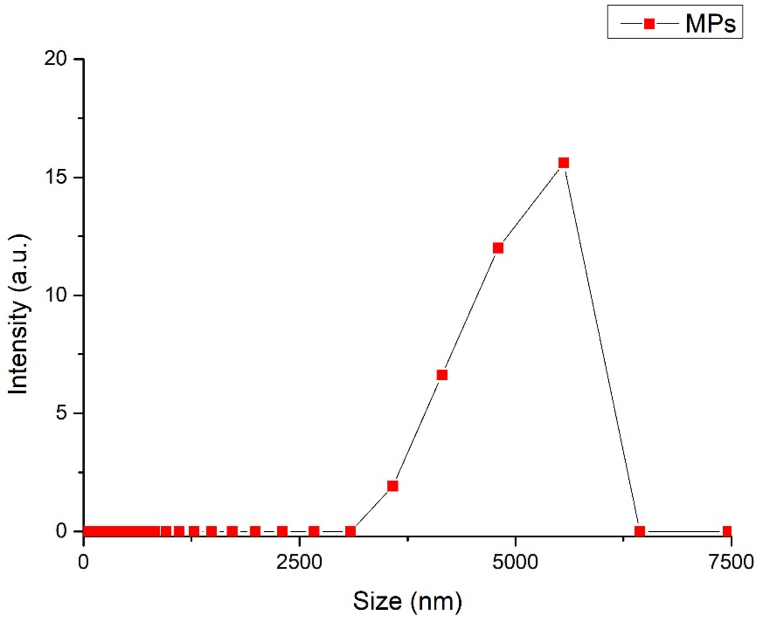

Figure S8. Size measurement of 4 μm MPs by Dynamic Light Scattering at outlet#1 when 30 V were applied at the electrodes.

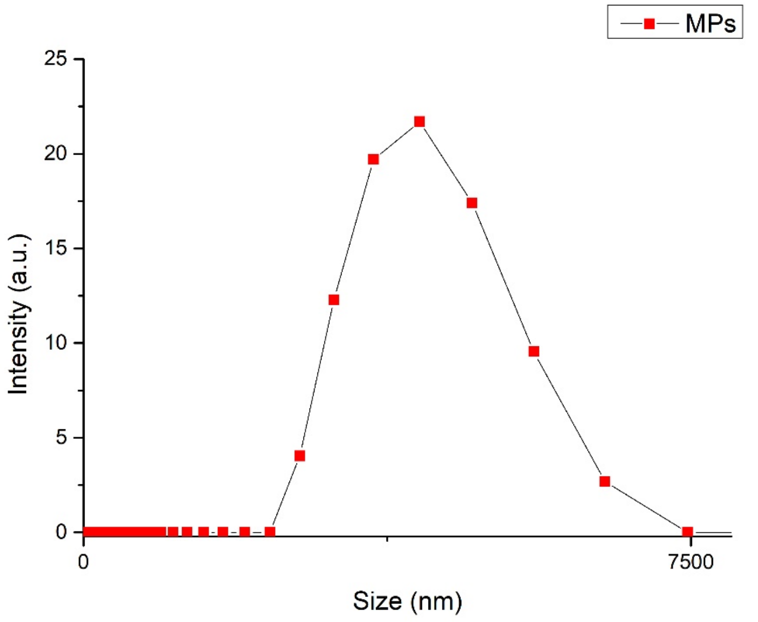

Figure S9. Size measurement of 4 μm MPs by Dynamic Light Scattering at outlet#1 when 40 V were applied at the electrodes.

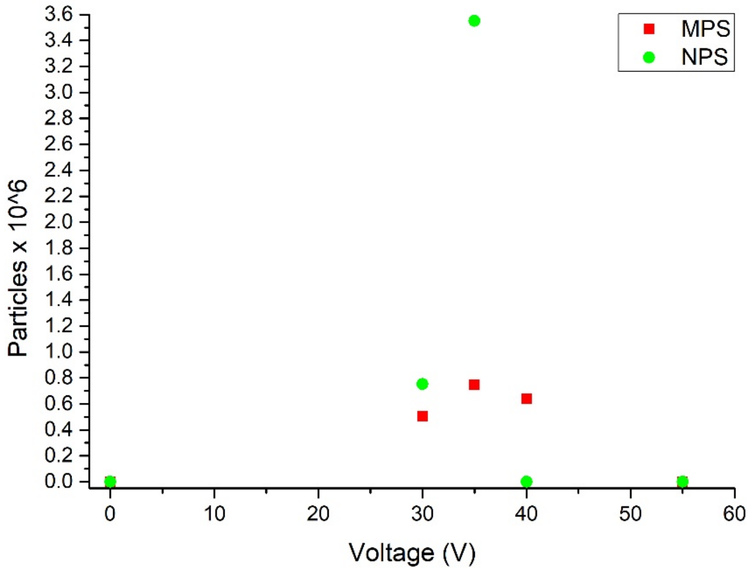

Figure S10. Number of M/NPs collected at outlet#2 when ΔV = 0, 30, 35, 40 and 55 V were applied at the electrodes.

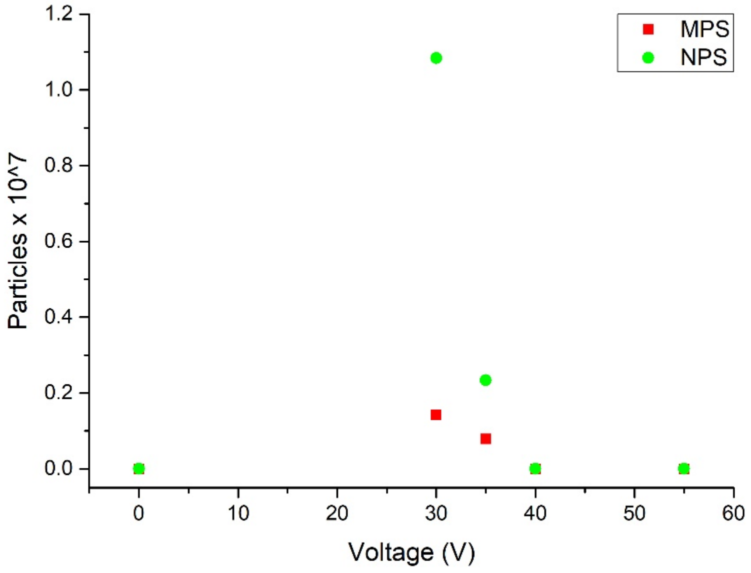

Figure S11. Number of M/NPs collected at outlet#3 when ΔV = 0, 30, 35, 40 and 55 V were applied at the electrodes.

Table S1. DLS particles measurement.

Sample	Electrophoretic mobility (μm·cm/V·s)	Conductivity (mS/cm)
MPs	14.9 ± 0.7	−0.9 ± 0.2
NPs	3.9 ± 0.6	−1.0 ± 0.8
EXs	13.6 ± 0.9	−0.7 ± 0.1
